# Evaluation of the HPA Axis’ Response to Pharmacological Challenges in Experimental and Clinical Early-Life Stress-Associated Depression

**DOI:** 10.1523/ENEURO.0222-20.2020

**Published:** 2021-01-13

**Authors:** Nayara Cobra Barreiro Barroca, Cristiane Von Werne Baes, Camila Maria Severi Martins-Monteverde, Nayanne Beckmann Bosaipo, Marcia Santos da Silva Umeoka, Julian Tejada, José Antunes-Rodrigues, Margaret de Castro, Mario Francisco Juruena, Norberto Garcia-Cairasco, Eduardo Henrique de Lima Umeoka

**Affiliations:** 1 Neuroscience and Behavioral Sciences Department, Ribeirão Preto School of Medicine, University of São Paulo, Ribeirão Preto, 14040-900, Brazil; 2 Physiology Department, Ribeirão Preto School of Medicine, University of São Paulo, Ribeirão Preto, 14040-900, Brazil; 3 Research Group on Neurobiology of Behavior, Cognition and Emotions, Faculty of Medicine, University Center Unicerrado, Goiatuba, 75600-000, Brazil; 4 Psychology Department, Federal University of Sergipe, São Cristóvão, 49100-000, Brazil; 5 Department of Psychological Medicine, Kings College London, London, SE5 8AF, United Kingdom

**Keywords:** depression, early-life stress, glucocorticoid receptor, HPA axis, mineralocorticoid receptor, translational psychiatry

## Abstract

Early-life stress (ELS) is associated with a higher risk of psychopathologies in adulthood, such as depression, which may be related to persistent changes in the hypothalamic-pituitary-adrenal (HPA) axis. This study aimed to evaluate the effects of ELS on the functioning of the HPA axis in clinical and experimental situations. Clinically, patients with current depressive episodes, with and without ELS, and healthy controls, composed the sample. Subjects took a capsule containing placebo, fludrocortisone, prednisolone, dexamethasone or spironolactone followed by an assessment of plasma cortisol the morning after. Experimentally, male Wistar rats were submitted to ELS protocol based on variable, unpredictable stressors from postnatal day (PND)1 to PND21. On PND65 animals were behaviorally evaluated through the forced-swimming test (FST). At PND68, pharmacological challenges started, using mifepristone, dexamethasone, spironolactone, or fludrocortisone, and corticosterone levels were determined 3 h after injections. Cortisol response of the patients did not differ significantly from healthy subjects, regardless of their ELS history, and it was lower after fludrocortisone, prednisolone, and dexamethasone compared with placebo, indicating the suppression of plasma cortisol by all these treatments. Animals exposed to ELS presented altered phenotype as indicated by an increased immobility time in the FST when compared with control, but no significant long-lasting effects of ELS were observed on the HPA axis response. Limitations on the way the volunteers were sampled may have contributed to the lack of ELS effects on the HPA axis, pointing out the need for further research to understand these complex phenomena

## Significance Statement

Early-life stress (ELS) is associated with a higher risk of psychopathologies in adulthood, including depression, which seems related to persistent changes in the hypothalamic-pituitary-adrenal (HPA) axis. We evaluated the effects of ELS on the functioning of HPA axis combining clinical and experimental approaches, challenging glucocorticoid receptor (GR) and mineralocorticoid receptor (MR) receptors with agonists and antagonists. Cortisol response of depressive patients did not differ from healthy subjects, regardless of their ELS history. Animals submitted to ELS presented altered behavior, as increased immobility time in the forced-swimming test (FST), but no effects were observed in response to GR and MR challenges. It provides insights into the complexity of HPA axis-related mechanisms involved in the long-lasting consequences of ELS in both experimental animals and humans.

## Introduction

Chronic or severe stress are known as a relevant factor in the development of psychopathologies (Kendler et al., 2002; Godoy et al., 2018a). Particularly, early-life stress (ELS), such as childhood neglect; parental deprivation; lack of basic care; physical, sexual, and psychological violence (Heim et al., 2000; Cohen et al., 2001; Martins-Monteverde et al., 2019), have been shown as risk factors for depression in adulthood (Cohen et al., 2001; Heim and Nemeroff, 2001; Kendler et al., 2002). ELS can affect the clinical course and worsen the treatment outcome (Tunnard et al., 2014; Williams et al., 2016; Duncko et al., 2019) and can be related to disturbed development of brain structures during this neurodevelopmental window (Anisman et al., 1998; Andersen, 2003; Murmu et al., 2006; Lupien et al., 2009; Krugers et al., 2017).

Clinical researchers are interested in understanding the mechanisms by which ELS might be linked to depression and whether they are consistent with neurobiological alterations found in animal models (Heim et al., 2008). Animal models of ELS are diverse, and common ones includes chronic unpredictable stressors, maternal separation, early deprivation, and isolated rearing in rodents (Lyons et al., 2010; Kumar et al., 2011; Zhang et al., 2013; Loi et al., 2014; Godoy et al., 2018b), as well as models that leads to alteration in the pattern of maternal behavior, such as the limited bedding and nesting protocol (Rice et al., 2008; Molet et al., 2014; Walker et al., 2017). These animal models have provided clues about neural changes because of ELS, including sensitization of neuroendocrine, autonomic, and behavioral responses (Heim et al., 2008; Coley et al., 2019), many associated to abnormalities of the hypothalamic-pituitary-adrenal (HPA) axis (Ladd et al., 1999; Buschdorf and Meaney, 2016). Some authors have shown that chronically depressed patients with history of ELS are more likely to show HPA axis alterations, and characteristics such as age, re-exposure to stressors, and nature of the maltreatment are highly associated to the intensity and pattern of the HPA axis abnormality (Mello et al., 2007; Joëls and Baram, 2009).

Mineralocorticoid receptor (MR) and glucocorticoid receptor (GR) are shown to be altered in stress-related psychopathologies (Reul et al., 2000; Juruena et al., 2006, 2010, 2013; Carvalho et al., 2008). Depressed patients commonly have non-suppression of the HPA axis or impaired feedback inhibition by dexamethasone, a synthetic glucocorticoid that binds preferentially to the GR (Newport et al., 2004; Heim et al., 2008; Tyrka et al., 2008; Carpenter et al., 2009; Klaassens et al., 2009), indicating reduced sensitivity to corticosteroids, particularly of GR (Juruena et al., 2006, 2010; Contreras et al., 2007; Carvalho et al., 2008; Baes et al., 2012). Studies using different HPA axis suppression test, such as prednisolone, a synthetic glucocorticoid that binds both to the MR and the GR, have investigated the role of both MR and GR in depression. For example, in Juruena et al. (2013), it was investigated the combined GR/MR stimulation with just prednisolone, prednisolone and spironolactone, and spironolactone alone, and authors found a lack of MR response in treatment-resistant depression, suggesting that there is an MR malfunctioning, as a downregulation, or even a pharmacokinetics or pharmacodynamics effect.

Both prednisolone and the dexamethasone tests suppress plasma and salivary cortisol levels in healthy subjects, but prednisolone seems to present greater suppression of the axis, mainly on salivary cortisol (Pariante et al., 2002). Juruena and colleagues (Juruena et al., 2006, 2009, 2010) found that depressed patients showed lesser cortisol suppression than controls after the dexamethasone test, but similar cortisol suppression after prednisolone. The usually preferred interpretation of these findings is that depressed patients (or, specifically, that sample of depressed patients) show a selective impairment of GR sensitivity, probed by DEX, whereas MR sensitivity, additionally probed by prednisolone, is mostly retained (Juruena et al., 2006). Moreover, MR agonist and antagonist (fludrocortisone and spironolactone) have also been used as pharmacological challenges to assess especially the MR function.

Data are still controversial among studies. Lembke et al. (2013) demonstrated that depressive patients had higher levels of plasma cortisol and impairment of the HPA axis negative feedback in response to fludrocortisone. A different study found lower levels of plasma cortisol in depressive patients treated with fludrocortisone and escitalopram and that fludrocortisone accelerated the response to antidepressant treatment (Otte et al., 2010). Our previous results showed a positive correlation of the plasma cortisol levels after placebo and Childhood Trauma Questionnaire (CTQ) scores in the depressed patients and controls. Interestingly, there was a positive correlation between plasma cortisol and the severity of ELS in patients with ELS, and no correlation was found in patients without ELS and in controls (Baes et al., 2014). Since the characterization of the processes involving ELS and psychopathologies are still imprecise, we attempted to combine basic and clinical research to understand better the mechanisms underlying the link between adverse experiences in childhood and depression. We evaluated the effects of ELS on the functioning of the HPA axis and the putative role of GR and MR in clinical depression and depressive-like experimental conditions.

## Materials and Methods

### Clinical study

#### Subjects

The Research Ethics Committee approved the clinical protocols of the General Clinical Hospital, at the Ribeirão Preto School of Medicine, University of São Paulo (Protocol 14324/2011). A total of 52 subjects, aged from 18 to 65 years, including 26 healthy controls and 26 patients with a diagnosis of current depressive episodes, including unipolar and bipolar depressive disorders (American Psychiatric Association, 2014), participated in the study. Written informed consent was obtained from all subjects. All patients were interviewed by a psychiatrist using the Mini International Neuropsychiatric Interview (Sheehan et al., 1998) for confirmation of the diagnosis of depression. For assessment of the severity of depression, participants were interviewed using the 21-item GRID Hamilton Depression Rating Scale (Hamilton, 1960). Patients scored with at least 16 on the 21-item GRID-HAM-D were included, ensuring the inclusion of patients with moderate to severe clinical levels of depressive illness (Rush et al., 2006). For the positive history of ELS, depressed patients were divided into two groups. The first included those with ELS (*n* = 17, 65.4%), and the second included those without ELS (*n* = 9, 34.6%). The ELS group was assessed using the CTQ (Bernstein et al., 1994), a retrospective self-report questionnaire that investigates the history of abuse and neglect during childhood and can be applied to adolescents from 12 years to adults. Portuguese version was validated by Grassi-Oliveira et al. (2006, 2014). Patients assigned values of frequency to 28 assertive graduate issues related to childhood situations and items were rated on a Likert scale ranging from 1 (never) to 5 (very often). The scores ranged from 5 to 25 for each item and also contained a subscale of minimization/denial to identify individuals responding in a socially desirable manner. A cut point for ELS was determined when the stress experience happened before 18 years old and was at least moderate-severe or severe-extreme degrees according to CTQ.

It was not possible to test patients in a drug-free state. All 26 patients were taking medication during the assessment. The following situations were determined as exclusion criteria: patients hypersensitive to corticosteroids or steroid users, smokers with >25 cigarettes/d, viral illness during the preceding two weeks or significant physical illness (allergy, autoimmune disease, hypertension, hematologic, endocrine, pulmonary, renal, hepatic, gastrointestinal, or neurologic conditions), and with a history of alcohol or drug abuse/dependence. They were also excluded pregnant or lactating women and patients with an intellectual disability or with psychosis unrelated to their depressive disorder or with organic cause for their depression.

The healthy controls were recruited from the hospital staff, students and the local community via public advertisements. The subjects of the control group were physically healthy individuals, as assessed by a complete medical history and examination; they were not taking any psychotropic medication and had no history of hypersensitivity to corticosteroids. Healthy individuals were excluded if they had a personal history of psychiatric disorder or a history of ELS.

#### Clinical protocol

The clinical study used a single-blind, non-randomized, placebo-controlled, repeated-measures design. Before each study day, the subjects were instructed to take one capsule (at 10 P.M.), containing placebo, or fludrocortisone (0.5 mg), or prednisolone (5 mg), or dexamethasone (0.5 mg), or spironolactone (400 mg), followed by an assessment of plasma cortisol on the next day. Each drug was administered with an interval of 48 h. No alcohol, coffee, tea or meals were allowed after each capsule. Subjects were instructed not to perform exercises or engage in stressful activities right after drug intake.

Cortisol assessment consisted of an analysis of one plasma sample collected at 9 A.M. (11 h after drug administration). Blood samples were analyzed at the Endocrinology Laboratory of the General Clinical Hospital, of Ribeirão Preto School of Medicine, University of São Paulo, by radioimmunoassay (Castro and Moreira, 2003).

### Experimental study

#### Subjects

All the procedures were approved by the Animal Ethical Committee of the Ribeirão Preto School of Medicine of the University of São Paulo (Protocol 29/2016) and were all performed by the National Council for Animal Experimentation Control (CONCEA). Two-week pregnant female Wistar rats were acquired from the Vivarium of the University of São Paulo Campus of Ribeirão Preto, housed individually in acrylic cages (45 × 32 × 17 cm), with food and water *ad libitum*, maintained under controlled ventilation and temperature (25°C) under 12/12 h light/dark cycle (lights on at 7 A.M.). Rats were checked twice a day from the time they arrived at the animal facility of the Physiology Department until the day of birth [postnatal day (PND)0]. After birth, each litter was culled to eight pups, six males and two females, and litters were assigned in two groups: ELS group (ELS, *n* = 18 male pups), which were exposed to the ELS protocol described below, and control group (CTRL, *n* = 16 male pups) without stress exposure. The number of animals, as well as their suffering, was kept to the minimum necessary to achieve the objectives of the study.

#### Experimental protocol

ELS protocol started on PND1. The protocol consisted of daily exposure of the animals to multimodal stressors for 21 days (until PND21). The rats received one stressor per day, alternately, to avoid habituation to the stressors (Mineur et al., 2006; Godoy et al., 2018b), while animals from the control group were kept undisturbed in their home-cages all the time. The following stressors were applied. (1) Maternal and peer separation (Kumar et al., 2011; Zhang et al., 2013; Godoy et al., 2018b): animals were placed in clean cages covered with sawdust without visual or physical contact with their mother or siblings for 60 min. (2) Restraint and shaking (Godoy et al., 2018b): animals were placed in 50-ml Falcon tubes (until PND12) or acrylic restrainers (from PND12 to PND21) and then on top of a shaker at 30 rpm for 60 min. (3) Exposure to cold (Papp et al., 1991; Godoy et al., 2018b): animals were taken to a cold chamber at 4°C for 10 min. At PND21, animals were weaned. From PND21 until PND60, there was no manipulation of any of the groups, except for regular cleaning of the cages, twice a week. Behavioral phenotyping using the forced-swimming test (FST), started at PND65. The FST was performed in two sessions: training session of 15 min (900 s) and test session of 5 min (300 s), with a 24-h interval between sessions, as described by Slattery and Cryan (2012). In both sessions, the animals were individually placed into a transparent acrylic cylinder (35 cm in diameter × 60 cm height – 50-l volume), with a 35-cm water column, enough to avoid the rats to touch the bottom of the cylinder with their legs or tail. The water was changed and the cylinder sanitized after each trial. The water temperature was controlled at 25 ± 2°C. All sessions were recorded using a video camera, and the time spent on each behavior (climbing, immobility, active swimming, and diving) was assessed manually by an observer and recorded by means of the X-Plo-Rat 2005 software.

All behavioral analyses were performed blindly to experimental conditions. Immobility was considered when the animals remained floating and performing movements necessary only to maintain their heads or nostrils above the water surface. Climbing was considered when the animals exhibited vigorous movement and simultaneous extension of all limbs, exceeding the movements required to keep only the head above the water and generally trying pushing themselves out of the water. Diving was considered when the animals were completely submerged, exploring in diving the bottom of the apparatus. Finally, active swimming was considered when the animals explored the surface of the apparatus, actively swimming on the horizontal axis.

After the end of the behavioral testing, pharmacological challenges, using antagonists and agonists of GR and MR, were performed. Starting at PND68, rats from both groups were subcutaneously treated every other day (48-h interval) with vehicle (propylene glycol), fludrocortisone (5 mg/kg), dexamethasone (5 mg/kg), spironolactone (25 mg/kg), or mifepristone (25 mg/kg) at 5 P.M. All animals received all the drugs (repeated measures) with a 48-h interval between each drug administration. Three hours after each drug administration (8 P.M.), blood was collected via tail venesection for quantification of plasma corticosterone by radioimmunoassay, as previously described (Elias et al., 2002; Umeoka et al., 2011). We chose to collect the blood samples at 8 P.M. here (night, opposing the clinical protocol), to get the baseline during the peak of corticosterone release in rodents since they have inverted circadian rhythm (Itoh et al., 1981).

### Statistical analysis

As an effort to practice and promote better inference in neuroscience (Bernard, 2019; Calin-Jageman and Cumming, 2019), we conducted estimation statistics analyses to replace *t* tests, and in addition to ANOVAs in our quantitative results, therefore full data are visible in all our plots. Clinical analyses were conducted using the Statistical Package for Social Sciences, SPSS 20.0. Descriptive statistics were used for analysis of the sociodemographic; χ^2^ tests and one-way ANOVA with Bonferroni *post hoc* tests were applied for clinical characteristics of the samples and the scores of psychiatric evaluation instruments. Two-way ANOVA was applied to determine main variable effects, interaction, and for within-groups comparisons of the plasma cortisol measurements after pharmacological challenges. Estimation statistics analysis was conducted to assess the mean difference between groups, 95% confidence intervals (95%CIs) and two-sided permutation *t* test *p* values.

Animal behavior data were analyzed through the so-called Gardner–Altman two-groups estimation plot, the estimation statistics alternative to Student’s *t* test. For plasma corticosterone measurements after pharmacological challenges, two-way ANOVA was applied, using the Statistical Package GraphPad Prism 6.1, to determine main effects and interaction of the variables. Multi two-group Cumming plot was applied to assess the mean difference between groups, 95%CIs and two-sided permutation *t* test *p* values.

Estimation statistics were performed through an online tool (https://www.estimationstats.com/; Ho et al., 2019) and using the DaBest package for Python (Ho et al., 2019) downloaded from GitHub repository (https://github.com/ACCLAB/DABEST-python) and customized in house by J.T. and made a fork available at https://github.com/julian-tejada/DABEST-python/. Therefore, here we also report and plot mean differences between groups with 95%CIs and two-sided permutation *t* test *p* values.

## Results

### Clinical study

#### Clinical characterization of the sample

There were no differences among patients with or without ELS and controls in gender (χ^2^2,0 = 2.18, *p* = 0.33), body mass index (BMI; *F*_(2,0)_ = 0.43, *p* = 0.71), and age (*F*_(2,0)_ = 0.34, *p* = 0.65). The use of psychotropic medications, either selective serotonin reuptake inhibitors and tricyclic antidepressants, as well as other antidepressants, antipsychotics, mood stabilizers, and benzodiazepines, was also similar between patients with and without ELS. We also did not find difference regarding the polarity of depression and previous history of suicide attempt in both depressive groups as well as in the contraceptive use and menopause among depressive women with and without ELS, none of them used hormone replacement therapy, as shown in Table 1.

**Table 1:** Sociodemographic characteristics of depressive patients with and without ELS and controls

	Depressed patients		Controls (*n *=* *26)	Statistics	*p*
	With ELS (*n *=* *17)	Without ELS (*n *=* *9)			
Gender, *n* (%)				χ^2^ (df) = 2.18 (2.0)	0.33
Female	13 (76.5)	7 (77.8)	15 (57.7)		
Male	4 (23.5)	2 (22.2)	11 (42.3)		
Age, years (±SEM)	35.9 (±4.3)	36.3 (±3.0)	33.3 (±1.8)	*F* (df) = 0.43 (2.0)	0.65
BMI, kg/m^2^ (±SEM)	27.8 (±3.2)	26.7 (±1.6)	25.8 (±0.9)	*F* (df) = 0.34 (2.0)	0.71
Smoking, *n* (%)	2 (11.8)	0 (0)	2 (7.7)	χ^2^ (df) = 1.14 (2.0)	0.56
Diagnostic, *n* (%)				χ^2^ (df) = 0.47 (1.0)	0.41
Unipolar depression	11 (64.7)	7 (77.8)	-		
Bipolar depression	6 (35.3)	2 (22.2)	-		
Medication, *n* (%)					
SSRI	12 (70.6)	6 (66.7)	-	χ^2^ (df) = 0.04 (1.0)	0.58
TCA	4 (23.5)	2 (22.2)	-	χ^2^ (df) = 0.006 (1.0)	0.67
Others AD	2 (11.8)	2 (22.2)	-	χ^2^ (df) = 0.49 (1.0)	0.43
Antipsychotics	6 (35.3)	5 (55.6)	-	χ^2^ (df) = 0.99 (1.0)	0.28
Mood stabilizer	5 (29.4)	5 (55.6)	-	χ^2^ (df) = 1.96 (1.0)	0.19
Benzodiazepine	12 (70.6)	3 (33.3)	-	χ^2^ (df) = 3.34 (1.0)	0.07
Attempted suicide, *n* (%)	12 (70.6)	4 (44.4)	-	χ^2^ (df) = 1.69 (1.0)	0.18
CTQ total, mean (±SEM)	68.3 (±4.6)^#¶^	35.6 (±1.5)	30.2 (±0.9)	*F* (df) = 57.56 (2.0)	<0.001
GRID-HAM-D_21_, mean (±SEM)	28.6 (±1.7)^¶^	25.3 (±1.4)^¶¶^	1.8 (±0.3)	*F* (df) = 198.23 (2.0)	<0.001
*N *=* *35 (100%)					
Menopause, *n* (%)	3 (23.1)	3 (42.9)	1 (6.7)	χ^2^ (df) = 4.02 (2.0)	0.13
CP, *n* (%)	6 (46.2)	4 (57.1)	5 (33.3)	χ^2^ (df) = 1.19 (2.0)	0.55

*N *=* *52 (100%). BMI: body mass index; SSRIs: selective serotonin reuptake inhibitors; TCA: tricyclic antidepressants; AD: antidepressants; CTQ: Childhood Trauma Questionnaire; GRID-HAM-D_21_: GRID-Hamilton depression rating scale; CP: contraceptive pills; #*p* < 0.05 between depressed patients with ELS and depressed patients without ELS; ¶*p* < 0.05 between depressed patients with ELS and controls and ¶¶*p* < 0.05 between depressed patients without ELS and controls.

The difference in the severity of ELS among the three groups was detected by the total CTQ score (*F*_(2,0)_ *=* 57.56, *p* < 0.001). The Bonferroni *post hoc* test showed that patients with ELS presented higher total CTQ scores when compared with patients without ELS (*p* < 0.001) and control group (*p* < 0.001), with no difference between patients without ELS and control group (*p* = 0.45). The scores for depression, evaluated by the HAM-D_21_ scale symptoms, revealed differences among all groups (*F*_(2,0)_ *=* 198.23, *p* < 0.001). Bonferroni *post hoc* test revealed that, while no significant differences were found between patients with and without ELS in the severity of depressive symptoms (*p* = 0.21), both groups had higher scores compared with controls (*p* < 0.001 and *p* < 0.001; Table 1).

#### Effects of GR and MR agonists and antagonists on plasma cortisol levels in depressed patients with and without ELS and controls

**Figure 1.**
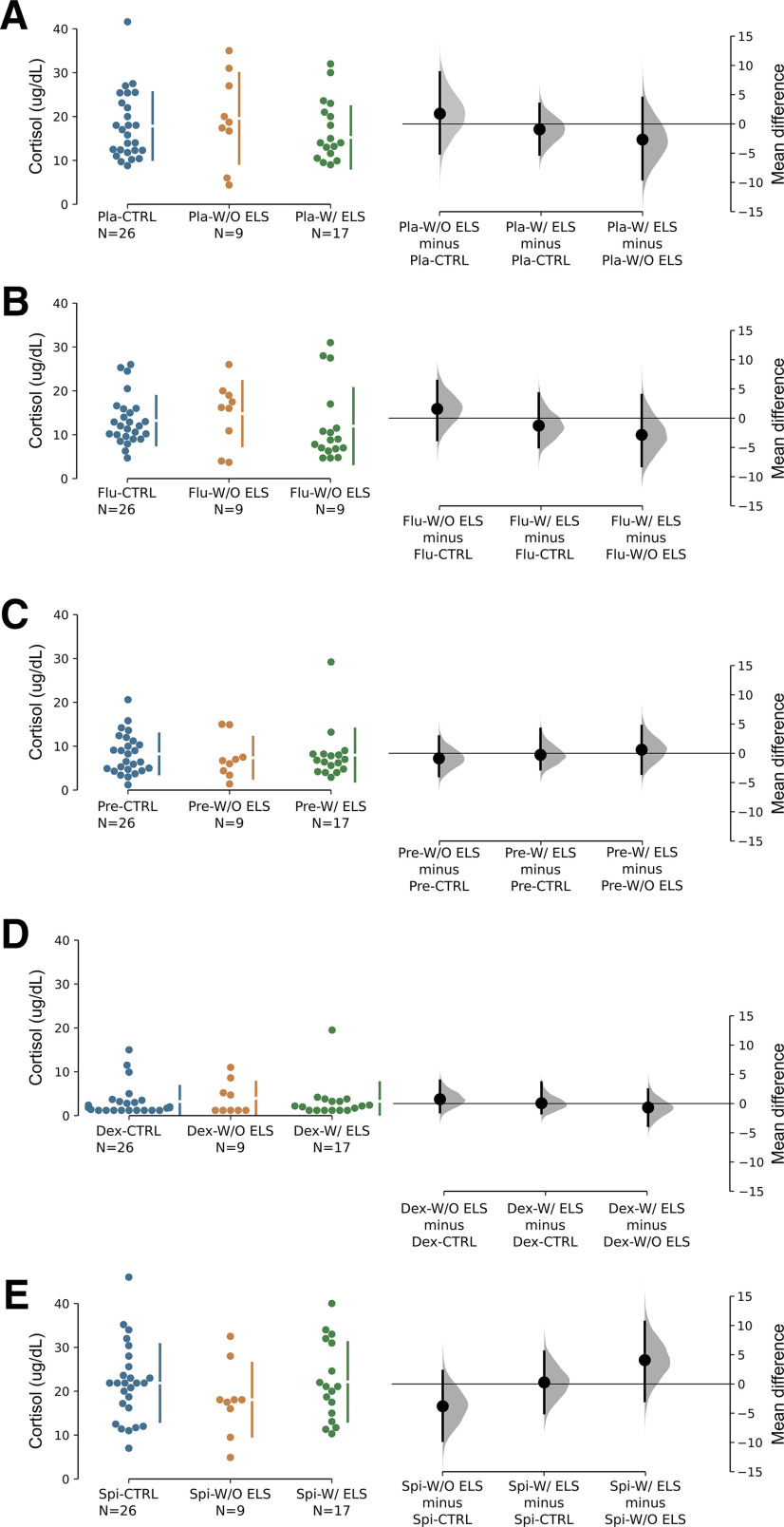
Plasma cortisol concentration (μg/dl) after administration of placebo (***A***), fludrocortisone (***B***), prednisolone (***C***), dexamethasone (***D***), and spironolactone (***E***) in depressive patients with and without ELS and controls. The mean difference for the thrtee within-treatment comparisons (CTRL vs W/O ELS, CTRL vs W/ELS, and W/O ELS vs W/ELS) are shown in the estimation plot. The raw data are plotted on the left-hand side of the panel; each mean difference is plotted on the right-hand side of the panel as a bootstrap sampling distribution. Mean differences are depicted as big black dots and the 95%CIs are indicated by the ends of the vertical error bars. Compared with placebo treatments with fludrocortisone, prednisone, dexamethasone, but not spironolactone, were able to suppress the cortisol response.

The pharmacological challenges affected the plasma cortisol levels (two-way ANOVA, *F* = 39.05; df = 4.0; *p* < 0.001). We found lower cortisol levels in fludrocortisone-treated (*p* < 0.001), prednisolone-treated (*p* < 0.001), and dexamethasone-treated (*p* < 0.001), but not spironolactone-treated (*p* = 0.30), groups compared with placebo, indicating a suppression of the HPA axis by these treatments. No interaction groups-challenges, nor group effects were found (*F* = 0.66, df = 8.0, *p* = 0.72 and *F* = 0.05, df = 2.0, *p* = 0.95, respectively). The unpaired effect sizes mean differences between the three groups, 95%CIs as well as the *p* values of the two-sided permutation *t* test were obtained, for each treatment, through estimation statistics analyses and are shown below on Table 2 and plotted on Figure 1.

**Table 2:** Plasmatic cortisol levels of controls and depressive patients with and without ELS

Comparisons	CTRL-W/O ELS		CTRL-W/ ELS		W/O ELS-W/ELS	
Treatments	Mean difference [95%CI]	*p*	Mean difference [95%CI]	*p*	Mean difference [95%CI]	*p*
Placebo	1.75 [95%CI −5.05, 8.81]	0.585	−0.931 [95%CI −5.23, 3.45]	0.702	−2.68 [95%CI −9.48, 4.46]	0.432
Fludrocortisone	1.58 [95%CI −3.74, 6.38]	0.501	−1.27 [95%CI −4.97, 4.23]	0.558	−2.86 [95%CI −8.2, 3.95]	0.411
Prednisolone	−0.869 [95%CI −3.9, 2.88]	0.633	−0.263 [95%CI −2.75, 4.18]	0.878	0.606 [95%CI −3.51, 4.67]	0.829
Dexamethasone	0.736 [95%CI −1.48, 3.91]	0.599	0.0566 [95%CI −1.66, 3.61]	0.966	−0.68 [95%CI −3.81, 2.4]	0.756
Spironolactone	−3.81 [95%CI −9.7, 2.22]	0.268	0.268 [95%CI −4.99, 5.54]	0.919	4.07 [95%CI −2.9, 10.6]	0.269

Mean differences, 95%CIs and *p* values of two-sided permutation *t* test from group comparisons (CTRL, with (W/) ELS and without (W/O) ELS) for every HPA axis challenge obtained from estimation statistics analyses.

### Experimental study

#### Effects of ELS on the FST

**Figure 2.**
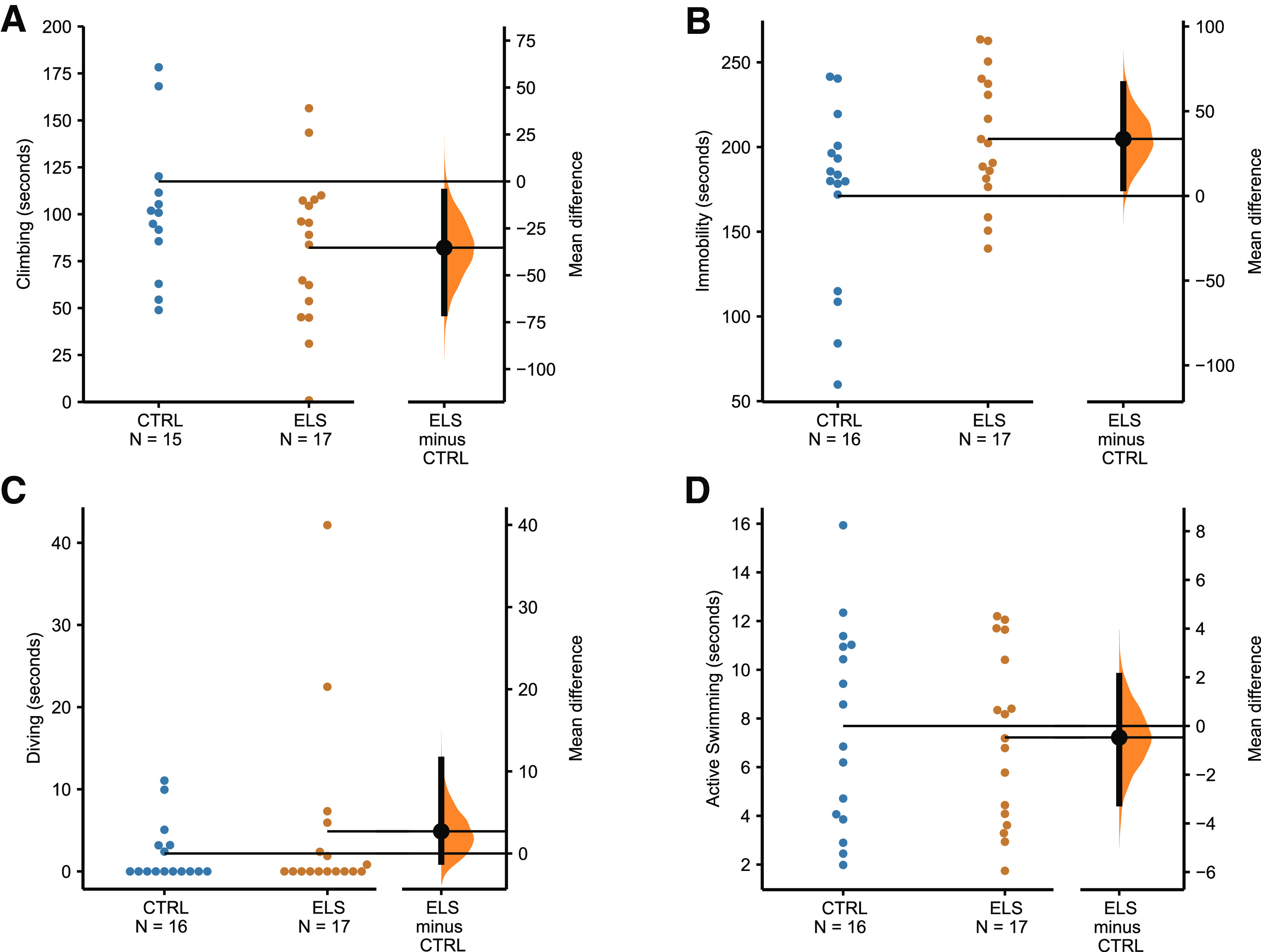
The average percentage of total time spent in (***A***) climbing, (***B***) immobility, (***C***) diving, or (***D***) active swimming during the test session (300 s) of the forced swim test. The mean differences between CTRL and ELS for every behavior are shown in the Gardner–Altman plot. The raw data are plotted on the upper axes; the mean difference is plotted on the lower axes as a bootstrap sampling distribution. Mean differences are depicted as big black dots and the 95%CIs are indicated by the ends of the vertical error bars.

Compared with the control group ELS was effective in decreasing climbing time, the unpaired mean difference was −35.3 with 95%CI of −70.2 to −5.53 (*p* = 0.0412; Fig. 2*A*), as well as in increasing the immobility time with a mean difference of 33.6 and 95%CI of 4.52–66.0 (*p* = 0.0444; Fig. 2*B*). No significant differences were observed in diving (2.7 [95.0%CI −1.01, 11.4], *p* = 0.429; Fig. 2*C*) and active swimming (−0.469 [95.0%CI −3.18, 2.06], *p* = 0.725; Fig. 2*D*) behaviors.

#### Effects of GR and MR agonists and antagonists on plasma corticosterone levels in rats submitted to ELS protocols

**Figure 3.**
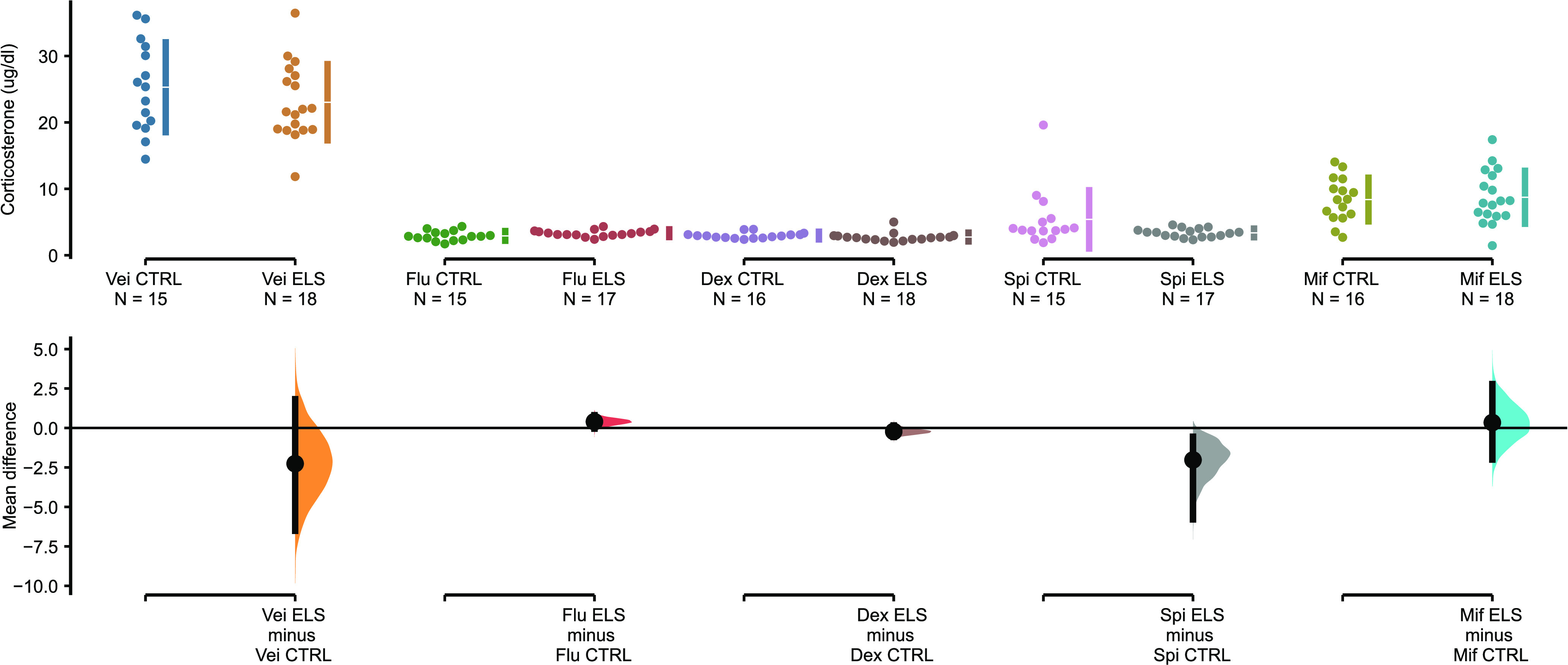
Plasma corticosterone concentration (μg/dl), 3 h after administration of vehicle (propylene glycol), fludrocortisone, dexamethasone, spironolactone, or mifepristone in ELS or control animals. The mean differences between CTRL and ELS for each treatment are shown in the Cumming plot. The raw data are plotted on the upper axis; the mean differences are plotted on the lower axis as a bootstrap sampling distribution. Mean differences are depicted as big black dots and the 95%CIs are indicated by the ends of the vertical error bars.

Pharmacological challenges decreased the levels of plasma corticosterone compared with the vehicle group (*F*_(4,155)_ = 212.7; *p* < 0.0001). However, no effect of ELS (*F*_(1,15)_ = 1.873, *p* = 0.17) or interaction between factors (*F*_(4,155)_ = 1.108, *p* = 0.35) were found. All treated groups, regardless of ELS exposure, presented lower plasma corticosterone concentration than the group receiving the vehicle. Significant difference in corticosterone levels between ELS and control groups was observed only after spironolactone treatment. The unpaired mean difference between control and ELS, 95%CI and two-sided permutation *t* test *p* value, revealed by the estimation statistics, for vehicle treatment was −2.26 [95.0%CI −6.52, 1.82] *p* = 0.298; for fludrocortisone was 0.399 [95.0%CI −0.042, 0.807] *p* = 0.0748; for dexamethasone was −0.22 [95.0%CI −0.571, 0.156] *p* = 0.285; for spironolactone was −2.02 [95.0%CI −5.79, −0.553] *p* = 0.0204 and for mifepristone was 0.343 [95.0%CI −2.0, 2.78] *p* = 0.77 (Fig. 3). To address a possible correlation between depressive-like behavior (immobility time on the FST) and HPA axis challenges outcome (plasmatic corticosterone levels) we performed Pearson’s correlation test, which revealed no significant correlations between these two variables after all treatment except mifepristone in the ELS group and all treatments in the control (data not shown).

## Discussion

In the present study, we explored in the clinical setting and an animal model, whether ELS exerts influences on behavioral parameters and the responsiveness of the HPA axis, challenging the function of GR and MR. It was found in the clinical scenario, that depressed patients, with and without ELS history, as well as non-depressed subjects, had similar suppressive responses on plasma cortisol levels after pharmacological challenges. Similarly, the animal study showed that ELS led to altered behavior in adulthood, but no differences in suppression plasma corticosterone levels was observed compared with control group. The dysregulation of the HPA axis has been associated with many psychiatric disorders, including hypersecretion of CRH, ACTH, and cortisol or hypoactivation of the HPA axis, marked by a blunted HPA axis response (Stetler and Miller, 2011). In a previous study, our group found, in a smaller sample of depressive patients with a history of ELS, a positive correlation of plasma cortisol levels and the CTQ score after placebo. This finding was also observed in controls. It was also observed a positive correlation between plasma cortisol and the severity of the depression (Baes et al., 2014).

It is worth keeping in mind that although not completely understood, and because they are very complex by themselves, the HPA axis and corticosteroids are part of a much more intricate system (Joëls and Baram, 2009). There are many other alternative mechanisms which might interact and contribute to ELS underlying depression. Beyond the classic CORT and ACTH stress hormones, we cannot neglect the regulatory role of the hypothalamic hormones oxytocin (Oxt) and arginine vasopressin (AVP) in the stress sensitivity, for example, related to the impacts of chronic stress in their receptors (Lesse et al., 2017). Also, changes in neural circuits such as those related to the reward system can be compromised, as discussed in the recent review by Birnie et al. (2020). Some pathways within the reward circuitry are especially vulnerable to suffer impacts from early-life stressors, such as altered CRH expression and function, which intensifies connectivity of nucleus accumbens with structures of fear/anxiety or related to detection, processing and retrieval of emotional events, through the amygdala, therefore disrupting the function of pleasure and reward (Birnie et al., 2020). We cannot also forget that the ELS has an additional impact at altering the epigenome, for example, through methylation of the GR gene promoter in the hippocampus, histone acetylation and transcription factor (NGFI-A) binding to the GR promoter, all together having important implications in stress response and consequences (Weaver et al., 2004; Klengel and Binder, 2015).

Some studies found no differences in basal plasma cortisol levels between depressive and control patients (Levitan et al., 2002; Gervasoni et al., 2004; Pintor et al., 2007), mainly when ELS was not considered. However, most of the studies have shown an increased in HPA activity in depressed patients (de Winter et al., 2003; Carroll et al., 2007; Contreras et al., 2007; Paslakis et al., 2011). When using different methods of assessing cortisol levels, Gerritsen et al. (2019) found no difference in hair cortisol between depressive patients and controls and Høifødt et al. (2019) detected that elevated salivary evening cortisol, but not morning cortisol, was significantly related to depressive symptoms. Also, Horst et al. (2019) demonstrated that patients with recurrent depression presented lower baseline but higher salivary cortisol awakening response than controls. Regarding studies on individuals with ELS (Newport et al., 2004; Carpenter et al., 2007), similarly to our current study, no differences were found in the baseline levels of plasma cortisol in individuals with and without ELS. Recently, Spitzer et al. (2018) determined that free salivary cortisol responses were not significantly different between women with or without major depression and with or without ELS history. Duncko et al. (2019) found that within depressed patients, those with a history of ELS presented lower cortisol concentration when compared with those with no such history, although they have used hair cortisol, and not plasma cortisol, to assess it.

A variety of methodology has been used to study the HPA function (Juruena et al., 2018). There are many studies on depressive patients with ELS after dexamethasone and Dex/CRH tests with still unclear results. Decreased plasma cortisol levels and a suggested increased GR activity have been demonstrated in individuals with ELS (Newport et al., 2004; Carpenter et al., 2009; Klaassens et al., 2009). Other studies showed no cortisol suppression after the Dex/CRH test in individuals with ELS, suggesting decreased GR activity in these patients (Heim et al., 2008; Tyrka et al., 2008). According to Tyrka et al. (2008), individuals who went through a prolonged parental separation, abandonment, or parental death during childhood also present an increased plasma cortisol response to the Dex/CRH test. Heim et al. (2008) showed that men with ELS presented higher plasma cortisol levels after the Dex/CRH test when compared with non-abused men and this level could be correlated with the severity, duration, and earlier onset of the abuse. Although controversial, it seems to be an agreement that putative abnormalities in the GR activity in response to the Dex/CRH test are a characteristic of patients with ELS history. Significantly, because of the complexity of the cortisol response to the Dex/CRH test, in which two regulators of the HPA axis are involved, i.e., the HPA axis negative feedback mechanism and the HPA stress response, it is not possible to compare results from baseline hormonal findings with cortisol response to the Dex/CRH test.

Most of the studies regarding depressive patients with ELS and the HPA axis response are focused on HPA axis suppressibility by glucocorticoids leading to infer that the GR are mainly involved in the altered mechanisms. Only a few studies are looking at the MR functions. In the present study, it was used dexamethasone and prednisolone suppression tests, which act predominantly by binding to GR although they can also bind to MR. It was also used fludrocortisone test, which acts predominantly by binding to MR but part of its action is exerted by binding to the GR (Grossmann et al., 2004; Baes et al., 2014). In the same direction, spironolactone binds primarily to MRs, but it also has low affinity to GR and progesterone receptors (Schane and Potts, 1978; Rogerson et al., 2003). Indeed, we did not find a difference in cortisol response after dexamethasone, prednisolone, or fludrocortisone challenges among depressive patients with or without ELS and controls. However, one interesting result is the absence of difference in cortisol response observed after spironolactone or placebo, indicating that this specific pharmacological antagonist of the MR was not able to suppress the HPA axis. Looking at other hormones, for example, aldosterone (selective to MR), it would be interesting to get a better view about the MR sensitivity and function (Grossmann et al., 2004).

It is essential to consider some limitations of the clinical study. The sample size was relatively small, especially in the group of depressed patients without ELS. It was expected that most depressed patients would have ELS history since data show that most of the depressive episodes are preceded by psychosocial stress (Mello et al., 2009; Carr et al., 2013; Tunnard et al., 2014) or are associated with ELS (Norman et al., 2012; Shapero et al., 2014; Williams et al., 2016). However, a large number of patients would provide better insights, especially because ELS definition can be difficult and researchers have used diverse concepts and instruments to assess ELS (Straus et al., 1998; Bremner et al., 2000).

Since the patients were taking medication during the assessment, a drug-free state was impractical to assess in our sample, which can also be considered as a limitation for our interpretation of the results. However, it is interesting to note that previous studies had shown that there is no difference in the hormonal measures (cortisol levels after the Dex/CRH test) comparing patients under medication or not, independently of the type and number of antidepressant treatments during the index episode (Künzel et al., 2003; Kunugi et al., 2006). Carvalho et al. (2008) using an *in vitro* preparation showed that the antidepressant clomipramine reduces GR function in healthy subjects but not in depressive patients. Moreover, previous studies observed that antidepressants did not affect corticosteroid receptor function in depressed patients who were still unwell, as in our sample (Bauer et al., 2002, 2003; Cotter et al., 2002; Künzel et al., 2003).

It is interesting to note that it seems to be an association between the different subtypes of stressors experienced during childhood and the development, persistence and severity of psychopathology in adults (Carr et al., 2013; Chaby et al., 2017; Martins-Monteverde et al., 2019; Mazer et al., 2019). In that context, different subtypes of stressors can recruit different circuitries and lead to differential CORT responses, for example, when we refer to psychological or physical stressors, as demonstrated in experiments with rats using different stressors (maternal separation, cold exposure, propylene glycol injection, and restraint; Godoy et al., 2018b). When talking about clinical investigations, with CTQ, the instrument we choose to assess ELS, it is possible to evaluate five subtypes of childhood trauma: physical abuse, emotional abuse, sexual abuse, emotional neglect, and physical neglect (Bernstein et al., 2003; Grassi-Oliveira et al., 2014; Macedo et al., 2019; Martins-Monteverde et al., 2019; Mazer et al., 2019). This is a gold standard instrument, it has high test and retests reliability, and it is considered a reference in the international literature for the investigation of early stress (Wright et al., 2001; Grover et al., 2007; Sfoggia et al., 2008), although there are many other instruments, some of which include even other forms of ELS (Fink et al., 1995; Straus et al., 1998; Bremner et al., 2000; Schwartz et al., 2005).

A limitation is that CTQ does not allow the distinction between the moment when the trauma occurred, the proximity to the aggressor, and the frequency of ELS. According to some authors, the experiences of abuse and neglect that occurred in the first years of life are particularly more harmful to mental health than those that occurred in other phases (Manly et al., 2001; Hildyard and Wolfe, 2002), and preclinical and review studies had also highlighted the relevance of the developmental phase, when the stressors are experienced, to later consequences (Lupien et al., 2009; Bock et al., 2014; Arp et al., 2016; Miragaia et al., 2018). Another essential factor to be considered is the degree of proximity to the aggressor, that is, the relationship that the victim had with the abuser (Pompili et al., 2009). In this sense, some studies have suggested that the evaluation of specific forms of ELS is more reliable than global assessments (Brewin et al., 1993).

Given the limitations presented in the clinical study, it was applied a similar design using an animal model. It is interesting to note that results on the glucocorticoid suppression tests were similar in the primary and clinical approaches. It is well established that the development of psychopathologies in humans is related to individual differences associated with both vulnerability and resilience. Animal models enable better enlightenment of contributing factors to these differences (Armario and Nadal, 2013) by isolating variables. Chronic unpredictable stress model, which uses animal exposure to different kinds of stressors over a certain period, tries to mimic human situations, by presenting stressors with high unpredictability, uncontrollability, and moderated intensity (Armario and Nadal, 2013). The importance of these models relates to the chronic depressive model of gradual response to stress development over time (Duman, 2010). The multimodal ELS used in our current study had been previously characterized and validated as a no habituation stress model and has been demonstrated to induce physiological and behavioral changes, including later in life “depressive-like” behavior (Godoy et al., 2018b).

Our data on animals submitted to ELS showed decreased climbing and increased immobility time with no difference in diving and active swimming behaviors in the FST. Our findings are congruent with what many authors still consider as a readout for depressive-like behavior (Molendijk and de Kloet, 2019). The FST was initially developed to investigate the efficacy of potential antidepressant drugs (Porsolt et al., 1977); later, it has been used to evaluate phenotype depressive-like behaviors, but its interpretation has been recently questioned (Duman, 2010; Bogdanova et al., 2013; De Kloet and Molendijk, 2016; Molendijk and de Kloet, 2019). The increased FST immobility in animals previously submitted to ELS has been considered a depressive-type behavior, because it was associated with a passive coping strategy, rather than dealing effectively with a difficult situation (Porsolt et al., 1977). However, increased immobility in response to chronic stress can be a consequence of increased stress sensitivity, which may promote memory consolidation, as behavioral adaptation related to cope with inescapable stressors rather than a depressive phenotype (Molendijk and de Kloet, 2015, 2019; De Kloet and Molendijk, 2016).

Similar to a human study, in the animal model study, our data also showed a decreased plasma corticosterone levels after pharmacological challenges with agonists and antagonists of GR and MR with no difference between ELS and control groups in any of the treatments. Corticosterone is a useful parameter to assess the HPA axis reactivity, but the levels after ELS are not consistent, especially when assessed later in life (Anisman et al., 1998; Naninck et al., 2015). Unpublished data from our laboratory also showed no effect on corticosterone levels in mice at baseline or after acute stress, but they show age-dependent interaction effects with regard to MR and GR mRNA expression after the ELS protocol. Lower expression of GR and MR after ELS was found in rats (Champagne et al., 2008) and also in primates (Arabadzisz et al., 2010), particularly in the hippocampus. Using the same ELS protocol, an increased stress-induced corticosterone secretion was observed after stressors at PND3, PND12, and PND21 but no difference in baseline 24 h after the stressor nor during adulthood at PND90 (Godoy et al., 2018b).

A limitation of our study is that 48-h intervals used between administrations of the different drugs might be not enough time for total drug elimination, which consequently could interfere with the effect of the subsequent administered drug, masking the effects after ELS. However, even for a vehicle or for the first administered drug (fludrocortisone, for the animal study), i.e., administration without risk of being influenced by previous administrations, no significant differences were found between the control and ELS groups. Our experiments were designed similarly from previous studies in the literature, which allows us to standardize the intervals between endocrine challenges in 48 h, reducing the variability between different cortisol assessments in the same individual (Pariante et al., 2002; Juruena et al., 2006, 2010, 2013; Baes et al., 2014). In humans, a study showed that prednisolone has a shorter half-life compared with dexamethasone, and no plasma levels of prednisolone were detected after 20 h of administration (Pariante et al., 2002). In animals, a study showed a half-life of ∼3 h for dexamethasone in female rats, after intravenous or intramuscular administration, and the same for pregnant rats and fetal circulation (Samtani and Jusko, 2005, 2007). According to the IBM Micromedex (electronic version; Micromedex, 2020) human-based database, fludrocortisone has an elimination half-life of 3.5 h; dexamethasone absorption of 10 min to 2 h and elimination half-life of 1.88–2.23 h; spironolactone elimination half-life of 1.4 h, and mifepristone absorption of 1–4 h and elimination half-life of 20 h to 85 h.

We should also consider a limitation of our study related to only using males in the experimental protocol. We understand the importance of including females, but for these experiments of depressive-like behavior and pharmacological challenges, they were not included. The use of females is increasingly encouraged for the scientific community, not only because of significant ethical aspects but also because the scientific interpretation of the results can significantly benefit from it. Using both males and females is especially relevant since neuropsychiatric disorders often manifest differently in men and women, either in incidence or in symptoms (Kessler et al., 2005; Novais et al., 2017); also, when talking about early experience, sex seems to be a significant contributor to the response to stressors (Bock et al., 2014; Loi et al., 2014; Walker et al., 2017; Bonapersona et al., 2019; Coley et al., 2019).

Also, a second hit is an important factor to be considered regarding the reaction to stressors, especially when evaluating psychopathologies and depression. In our experimental design, all animals underwent the FST test, as an attempt to isolate variables and only distinguish a group from the other by the ELS exposure, which may be seen as the FST working as a second stressor for the animals in the ELS group, or as a novel stressor for the animals in the CTRL group. Adverse experiences during early life can program the sensitivity, responsiveness, and reactivity to a second stressful event later in life, and some functional alterations because of ELS will only appear when combined with a subsequent second stress exposure (Lesse et al., 2017), which might be seen as a limitation to our interpretation of the ELS impact on behavioral response and HPA axis challenges. Despite that, we can say that the design can put this model closely related to kindling effect (Post, 2002; Juruena, 2014) or to the cumulative or two/multiple-hit hypothesis for the development of neuropsychiatric disorders, which presumes that neuropsychiatric conditions usually involves combination or interaction of two or more different stressors experienced in different lifespan points (Monroe and Harkness, 2005; Lesse et al., 2017).

In conclusion, our results on clinical and animal studies showing no differences in the plasma cortisol or corticosterone levels among depressive conditions with or without ELS and control subjects after different pharmacological challenges with GR and MR agonists and antagonists do not allow to conclude that the ELS leads to persistent changes in the HPA axis reactivity or not. It should be noticed that it is still not clear whether the HPA axis-related mechanisms are involved in the long-lasting consequences of ELS on the development of psychopathologies. Corticosteroids may act differently in melancholic or atypical depressive subtypes (Baes et al., 2014). Indeed, hypercortisolism has been related to melancholia and normal cortisol in atypical depression (Juruena et al., 2018). However, failure to observe changes in the level of corticosteroids does not mean that other central nervous system changes are not occurring. Alterations in other hormones such as CRH or ACTH levels, disbalance in neurotransmitters, altered neurogenesis or even GR and MR expression may be taking place (Joëls and Baram, 2009); although our data do not allow us to assess any of those. Also, there is highly conflicting literature in this field, and simplistic changes only in HPA axis and glucocorticoids levels might not be the best paths for potential new studies into ELS and depression relationship. HPA axis and glucocorticoids levels are only a portion of a very sophisticated and complex system (Joëls and Baram, 2009), which interact with alternative mechanisms (Birnie et al., 2020) and contribute to ELS underlying depression. Further studies are still necessary to understand the role of the HPA axis activity and the GR and MR to the contribution to different depressive conditions under ELS.

## Synthesis

Reviewing Editor: Aniko Korosi, University of Amsterdam Decisions are customarily a result of the Reviewing Editor and the peer reviewers coming together and discussing their recommendations until a consensus is reached. When revisions are invited, a fact-based synthesis statement explaining their decision and outlining what is needed to prepare a revision will be listed below. The following reviewer(s) agreed to reveal their identity: Jessica Bolton.

Reviewer 1

The current study is informative in that it attempt to parallel prior history of stress and current symptoms of depression with altered HPA function and responsively to pharmacological HPA challenge. It does so in both humans and rats, which is interesting. However, there are some technical challenges (counterbalancing) and timeline issues (e.g. timing of sampling differing between species) that impact the ability to draw direct comparisons. However, the data presented are still of value to the field, despite these drawbacks.

I applaud the authors for careful work and approach to this interesting topic. It is uncommon to attempt such a direct translational study employing both human and animal subjects. I appreciate the detailed response to the review comments and the modifications to the manuscript that have been made. While there remain some issues that impact the ability of the reader to directly relate these findings to one another (dosing, metabolism, timing of measures, and counterbalancing), the data are still valuable and will hopefully inform future work. I thank you for your contribution and your indulging the questions raised in the initial review.

Reviewer 2

This manuscript consists of an interesting “translational” study, or at least a comparison of a clinical study and results from an animal model that explore whether changes in the HPA axis may be relevant for early-life stress-induced depression. The (negative) findings reported here show that changes in the HPA axis and glucocorticoids may not underlie early-life stress-induced depression. There are a lot of conflicting results in this area, so this is an important finding to demonstrate that simplistic changes in glucocorticoids may not be a good focal point for future research in certain types of early-life stress and depression research.

I appreciate the authors’ incorporation of estimate statistics, as it is a nice addition to the paper.

I thank the authors for revising their manuscript; they have successfully addressed my concerns.

## References

[B1] American Psychiatric Association (2014) Manual diagnóstico e estatístico de transtornos mentais - DSM-5. ArtMed, Porto Alegre.

[B2] Andersen SL (2003) Trajectories of brain development: point of vulnerability or window of opportunity? Neurosci Biobehav Rev 27:3–18. 10.1016/s0149-7634(03)00005-8 12732219

[B3] Anisman H, Zaharia MD, Meaney MJ, Merali Z (1998) Do early-life events permanently alter behavioral and hormonal responses to stressors? Int J Dev Neurosci 16:149–164. 10.1016/s0736-5748(98)00025-2 9785112

[B4] Arabadzisz D, Diaz-Heijtz R, Knuesel I, Weber E, Pilloud S, Dettling AC, Feldon J, Law AJ, Harrison PJ, Pryce CR (2010) Primate early life stress leads to long-term mild hippocampal decreases in corticosteroid receptor expression. Biol Psychiatry 67:1106–1109. 10.1016/j.biopsych.2009.12.016 20132928

[B5] Armario A, Nadal R (2013) Individual differences and the characterization of animal models of psychopathology: a strong challenge and a good opportunity. Front Pharmacol 4:1–13. 24265618 10.3389/fphar.2013.00137 PMC3821037

[B6] Arp JM, Ter Horst JP, Loi M, den Blaauwen J, Bangert E, Fernández G, Joels M, Oitzl MS, Krugers HJ (2016) Blocking glucocorticoid receptors at adolescent age prevents enhanced freezing between repeated cue-exposures after conditioned fear in adult mice raised under chronic early life stress. Neurobiol Learn Mem 133:30–38. 27246249 10.1016/j.nlm.2016.05.009

[B7] Baes CVW, Tofoli SMC, Martins CMS, Juruena MF (2012) Assessment of the hypothalamic-pituitary-adrenal axis activity: glucocorticoid receptor and mineralocorticoid receptor function in depression with early life stress - a systematic review. Acta Neuropsychiatr 24:4–15. 10.1111/j.1601-5215.2011.00610.x 28183380

[B8] Baes CVW, Martins CMS, de Carvalho Tofoli SM, Juruena MF (2014) Early life stress in depressive patients: HPA axis response to GR and MR agonist. Front Psychiatry 5:1–12. 24478730 10.3389/fpsyt.2014.00002 PMC3900767

[B9] Bauer ME, Papadopoulos A, Poon L, Perks P, Lightman SL, Checkley S, Shanks N (2002) Dexamethasone-induced effects on lymphocyte distribution and expression of adhesion molecules in treatment-resistant depression. Psychiatry Res 113:1–15. 10.1016/s0165-1781(02)00243-3 12467941

[B10] Bauer ME, Papadopoulos A, Poon L, Perks P, Lightman SL, Checkley S, Shanks N (2003) Altered glucocorticoid immunoregulation in treatment resistant depression. Psychoneuroendocrinology 28:49–65. 10.1016/S0306-4530(02)00009-4 12445836

[B11] Bernard C (2019) Changing the way we report, interpret, and discuss our results to rebuild trust in our Research. eNeuro 6. 10.1523/ENEURO.0259-19.2019 PMC6709206 31453315

[B12] Bernstein DP, Fink L, Handelsman L, Foote J, Lovejoy M, Wenzel MA, Sapareto E, Ruggiero J (1994) Initial reliability and validity of a new retrospective measure of child abuse and neglet. Am J Psychiatry 151:1132–1136. 10.1176/ajp.151.8.1132 8037246

[B13] Bernstein DP, Stein JA, Newcomb MD, Walker E, Pogge D, Ahluvalia T, Stokes J, Handelsman L, Medrano M, Desmond D, Zule W (2003) Development and validation of a brief screening version of the childhood trauma questionnaire. Child Abus Negl 27:169–190. 10.1016/S0145-2134(02)00541-0 12615092

[B14] Birnie MT, Kooiker CL, Short AK, Bolton JL, Chen Y, Baram TZ (2020) Plasticity of the reward circuitry after early-life adversity: Mechanisms and significance. Biol Psychiatry 87:875–884. 32081365 10.1016/j.biopsych.2019.12.018 PMC7211119

[B15] Bock J, Rether K, Gröger N, Xie L, Braun K (2014) Perinatal programming of emotional brain circuits: an integrative view from systems to molecules. Front Neurosci 8:11. 10.3389/fnins.2014.00011 24550772 PMC3913903

[B16] Bogdanova OV, Kanekar S, D'Anci KE, Renshaw PF (2013) Factors influencing behavior in the forced swim test. Physiol Behav 118:227–239. 10.1016/j.physbeh.2013.05.012 23685235 PMC5609482

[B17] Bonapersona V, Damsteegt R, Adams ML, van Weert LT, Meijer OC, Joëls M, Sarabdjitsingh RA (2019) Sex-Dependent modulation of acute stress reactivity after early life stress in mice: relevance of mineralocorticoid receptor expression. Front Behav Neurosci 13:181. 10.3389/fnbeh.2019.00181 31440147 PMC6693524

[B18] Bremner JD, Vermetten E, Mazure CM (2000) Development and preliminary psychometric properties of an instrument for the measurement of childhood trauma: the early trauma inventory. Depress Anxiety 12:1–12. 10.1002/1520-6394(2000)12:1<1::AID-DA1>3.0.CO;2-W 10999240

[B19] Brewin CR, Andrews B, Gotlib IH (1993) Psychopathology and early experience: a reappraisal of retrospective reports. Psychol Bull 113:82–98. 10.1037/0033-2909.113.1.82 8426875

[B20] Buschdorf JP, Meaney MJ (2016) Epigenetics/programming in the HPA axis. Compr Physiol 6:87–110. 10.1002/cphy.c140027 26756628

[B21] Calin-Jageman RJ, Cumming G (2019) Estimation for better inference in neuroscience. eNeuro 6:1–11. 10.1523/ENEURO.0205-19.2019 PMC6709209 31453316

[B22] Carpenter LL, Carvalho JP, Tyrka AR, Wier LM, Mello AF, Mello MF, Anderson GM, Wilkinson CW, Price LH (2007) Decreased adrenocorticotropic hormone and cortisol responses to stress in healthy adults reporting significant childhood maltreatment. Biol Psychiatry 62:1080–1087. 10.1016/j.biopsych.2007.05.002 17662255 PMC2094109

[B23] Carpenter LL, Ross NS, Tyrka AR, Anderson GM, Kelly M, Price LH (2009) Dex/CRH test cortisol response in outpatients with major depression and matched healthy controls. Psychoneuroendocrinology 34:1208–1213. 10.1016/j.psyneuen.2009.03.009 19375869 PMC3580166

[B24] Carr CP, Martins CMS, Stingel AM, Lemgruber VB, Juruena MF (2013) The role of early life stress in adult psychiatric disorders: a systematic review according to childhood trauma subtypes. J Nerv Ment Dis 201:1007–1020. 10.1097/NMD.0000000000000049 24284634

[B25] Carroll BJ, Cassidy F, Naftolowitz D, Tatham NE, Wilson WH, Iranmanesh A, Liu PY, Veldhuis JD (2007) Pathophysiology of hypercortisolism in depression. Acta Psychiatr Scand 115:90–103. 10.1111/j.1600-0447.2007.00967.x 17280575

[B26] Carvalho LA, Juruena MF, Papadopoulos AS, Poon L, Kerwin R, Cleare AJ, Pariante CM (2008) Clomipramine in vitro reduces glucocorticoid receptor function in healthy subjects but not in patients with major depression. Neuropsychopharmacology 33:3182–3189. 10.1038/npp.2008.44 18368033 PMC3513411

[B27] Castro M, Moreira AC (2003) Análise crítica do cortisol salivar na avaliação do eixo hipotálamo-hipófise-adrenal. Arq Bras Endocrinol Metab 47:358–367. 10.1590/S0004-27302003000400008

[B28] Chaby LE, Zhang L, Liberzon I (2017) The effects of stress in early life and adolescence on posttraumatic stress disorder, depression, and anxiety symptomatology in adulthood. Curr Opin Behav Sci 14:86–93. 10.1016/j.cobeha.2017.01.001

[B29] Champagne DL, Bagot RC, Van Hasselt F, Ramakers G, Meaney MJ, De Kloet ER, Joëls M, Krugers H (2008) Maternal care and hippocampal plasticity: evidence for experience-dependent structural plasticity, altered synaptic functioning, and differential responsiveness to glucocorticoids and stress. J Neurosci 28:6037–6045. 10.1523/JNEUROSCI.0526-08.2008 18524909 PMC6670331

[B30] Cohen P, Brown J, Smaile E (2001) Child abuse and neglect and the development of mental disorders in the general population. Dev Psychopathol 13:981–999. 11771917

[B31] Coley EJ, Demaestri C, Ganguly P, Honeycutt JA, Peterzell S, Rose N, Ahmed N, Holschbach M, Trivedi M, Brenhouse HC (2019) Cross-generational transmission of early life stress effects on HPA regulators and Bdnf are mediated by sex, lineage, and upbringing. Front Behav Neurosci 13. 10.3389/fnbeh.2019.00101 PMC6521572 31143105

[B32] Contreras F, Menchon JM, Urretavizcaya M, Navarro MA, Vallejo J, Parker G (2007) Hormonal differences between psychotic and non-psychotic melancholic depression. J Affect Disord 100:65–73. 10.1016/j.jad.2006.09.021 17098292

[B33] Cotter PA, Mulligan OF, Landau S, Papadopoulos A, Lightman SL, Checkley SA (2002) Vasoconstrictor response to topical beclomethasone in major depression. Psychoneuroendocrinology 27:475–487. 10.1016/s0306-4530(01)00065-8 11912000

[B34] De Kloet ER, Molendijk ML (2016) Coping with the forced swim stressor: towards understanding an adaptive mechanism. Neural Plast 2016:1–13. 10.1155/2016/6503162 PMC4806646 27034848

[B35] de Winter RFP, Van Hemert AM, Derijk RH, Zwinderman KH, Frankhuijzen-Sierevoge AC, Wiegant VM, Goekoop JG (2003) Anxious-retarded depression: relation with plasma vasopressin and cortisol. Neuropsychopharmacology 28:140–147. 10.1038/sj.npp.1300002 12496950

[B36] Duman CH (2010) Models of depression. Vitam Horm 82:1–21. 10.1016/S0083-6729(10)82001-1 20472130

[B37] Duncko R, Fischer S, Hatch SL, Frissa S, Goodwin L, Papadopoulos A, Cleare AJ, Hotopf M (2019) Recurrence of depression in relation to history of childhood trauma and hair cortisol concentration in a community-based sample. Neuropsychobiology 78:48–57. 10.1159/000498920 30897568

[B38] Elias LLK, Campos AD, Moreira AC (2002) The opposite effects of short- and long-term salt loading on pituitary adrenal axis activity in rats. Horm Metab Res 34:207–211. 10.1055/s-2002-26711 11987031

[B39] Fink L, Bernstein DP, Handelsman L, Foote J, Lovejoy M (1995) Initial reability and validity of the childhood trauma interview. Am J Psychiatry 152:1329–1335. 7653689 10.1176/ajp.152.9.1329

[B40] Gerritsen L, Staufenbiel SM, Penninx BWJH, van Hemert AM, Noppe G, de Rijke YB, van Rossum EFC (2019) Long-term glucocorticoid levels measured in hair in patients with depressive and anxiety disorders. Psychoneuroendocrinology 101:246–252. 10.1016/j.psyneuen.2018.11.019 30472466

[B41] Gervasoni N, Bertschy G, Osiek C, Perret G, Denis R, Golaz J, Rossier MF, Bondolfi G, Aubry JM (2004) Cortisol responses to combined dexamethasone/CRH test in outpatients with a major depressive episode. J Psychiatr Res 38:553–557. 10.1016/j.jpsychires.2004.04.008 15458850

[B42] Godoy LD, Rossignoli MT, Delfino-Pereira P, Garcia-Cairasco N, Umeoka EH, de L (2018a) A comprehensive overview on stress neurobiology: basic concepts and clinical implications. Front Behav Neurosci 12:1–23. 30034327 10.3389/fnbeh.2018.00127 PMC6043787

[B43] Godoy LD, Umeoka EHL, Ribeiro DE, Santos VR, Antunes-Rodrigues J, Joca SRL, Garcia-Cairasco N (2018b) Multimodal early-life stress induces biological changes associated to psychopathologies. Horm Behav 100:69–80. 10.1016/j.yhbeh.2018.03.005 29548783

[B44] Grassi-Oliveira R, Stein LM, Pezzi JC (2006) Tradução e validação de conteúdo da versão em português do Childhood Trauma Questionnaire. Rev Saude Publica 40:249–255. 10.1590/s0034-89102006000200010 16583035

[B45] Grassi-Oliveira R, Cogo-Moreira H, Salum GA, Brietzke E, Viola TW, Manfro GG, Kristensen CH, Arteche AX (2014) Childhood Trauma Questionnaire (CTQ) in Brazilian samples of different age groups: findings from confirmatory factor analysis. PLoS One 9:e87118. 10.1371/journal.pone.0087118 24475237 PMC3903618

[B46] Grossmann C, Scholz T, Rochel M, Bumke-Vogt C, Oelkers W, Pfeiffer AFH, Diederich S, Bahr V (2004) Transactivation via the human glucocorticoid and mineralocorticoid receptor by therapeutically used steroids in CV-1 cells: a comparison of their glucocorticoid and mineralocorticoid properties. Eur J Endocrinol 151:397–406. 10.1530/eje.0.1510397 15362971

[B47] Grover KE, Carpenter LL, Price LH, Gagne GG, Mello AF, Mello MF, Tyrka AR (2007) The relationship between childhood abuse and adult personality disorder symptoms. J Pers Disord 21:442–447. 10.1521/pedi.2007.21.4.442 17685839 PMC4467781

[B48] Hamilton M (1960) A rating scale for depression. J Neurol Neurosurg Psychiatry 23:56–62. 10.1136/jnnp.23.1.56 14399272 PMC495331

[B49] Heim C, Nemeroff CB (2001) The role of childhood trauma in the neurobiology of mood and anxiety disorders: preclinical and clinical studies. Biol Psychiatry 49:1023–1039. 10.1016/s0006-3223(01)01157-x 11430844

[B50] Heim C, Newport DJ, Heit S, Graham YP, Wilcox M, Bonsall R, Miller AH, Nemeroff CB (2000) Pituitary-adrenal and automatic responses to stress in women after sexual and physical abuse in childhood. J Am Med Assoc 284:592–597. 10.1001/jama.284.5.592 10918705

[B51] Heim C, Newport DJ, Mletzko T, Miller AH, Nemeroff CB (2008) The link between childhood trauma and depression: insights from HPA axis studies in humans. Psychoneuroendocrinology 33:693–710. 10.1016/j.psyneuen.2008.03.008 18602762

[B52] Hildyard KL, Wolfe DA (2002) Child neglect: developmental issues and outcomes. Child Abus Negl 26:679–695. 10.1016/S0145-2134(02)00341-1 12201162

[B53] Ho J, Tumkaya T, Aryal S, Choi H, Claridge-Chang A (2019) Moving beyond P values: data analysis with estimation graphics. Nat Methods 16:565–566. 10.1038/s41592-019-0470-3 31217592

[B54] Høifødt RS, Waterloo K, Wang CEA, Eisemann M, Figenschau Y, Halvorsen M (2019) Cortisol levels and cognitive profile in major depression: a comparison of currently and previously depressed patients. Psychoneuroendocrinology 99:57–65. 10.1016/j.psyneuen.2018.08.024 30176378

[B55] Horst DM, Schene AH, Figueroa CA, Assies J, Lok A, Bockting CLH, Ruhé HG, Mocking RJT (2019) Cortisol, dehydroepiandrosterone sulfate, fatty acids, and their relation in recurrent depression. Psychoneuroendocrinology 100:203–212. 10.1016/j.psyneuen.2018.10.012 30388594

[B56] Itoh S, Katsuura G, Hirota R, Botan Y (1981) Circadian rhythm of plasma corticosterone in vagotomized rats. Experientia 37:380–381. 10.1007/BF01959873 7238816

[B57] Joëls M, Baram TZ (2009) The neuro-symphony of stress. Nat Rev Neurosci 10:459–466. 10.1038/nrn2632 19339973 PMC2844123

[B58] Juruena MF (2014) Early-life stress and HPA axis trigger recurrent adulthood depression. Epilepsy Behav 38:148–159. 10.1016/j.yebeh.2013.10.020 24269030

[B59] Juruena MF, Cleare AJ, Papadopoulos AS, Poon L, Lightman S, Pariante CM (2006) Different responses to dexamethasone and prednisolone in the same depressed patients. Psychopharmacology (Berl) 189:225–235. 10.1007/s00213-006-0555-4 17016711

[B60] Juruena MF, Pariante CM, Papadopoulos AS, Poon L, Lightman S, Cleare AJ (2009) prednisolone suppression test in depression: prospective study of the role of HPA axis dysfunction in treatment resistance. Br J Psychiatry 194:342–349. 10.1192/bjp.bp.108.050278 19336786

[B61] Juruena MF, Cleare AJ, Papadopoulos AS, Poon L, Lightman S, Pariante CM (2010) The prednisolone suppression test in depression: dose-response and changes with antidepressant treatment. Psychoneuroendocrinology 35:1486–1491. 10.1016/j.psyneuen.2010.04.016 20558006 PMC3513406

[B62] Juruena MF, Pariante CM, Papadopoulos AS, Poon L, Lightman S, Cleare AJ (2013) The role of mineralocorticoid receptor function in treatment-resistant depression. J Psychopharmacol 27:1169–1179. 10.1177/0269881113499205 23904409

[B63] Juruena MF, Bocharova M, Agustini B, Young AH (2018) Atypical depression and non-atypical depression: is HPA axis function a biomarker? A systematic review. J Affect Disord 233:45–67. 10.1016/j.jad.2017.09.052 29150144

[B64] Kendler KS, Sheth K, Gardner CO, Prescott CA (2002) Childhood parental loss and risk for first-onset of major depression and alcohol dependence: the time-decay of risk and sex differences. Psychol Med 32:1187–1194. 10.1017/s0033291702006219 12420888

[B65] Kessler RC, Berglund P, Demler O, Jin R, Merikangas KR, Walters EE (2005) Lifetime prevalence and age-of-onset distributions of DSM-IV disorders in the National Comorbidity Survey Replication. Arch Gen Psychiatry 62:593–602. 10.1001/archpsyc.62.6.593 15939837

[B66] Klaassens ER, van Noorden MS, Giltay EJ, van Pelt J, van Veen T, Zitman FG (2009) Effects of childhood trauma on HPA-axis reactivity in women free of lifetime psychopathology. Prog Neuropsychopharmacol Biol Psychiatry 33:889–894. 10.1016/j.pnpbp.2009.04.011 19389455

[B67] Klengel T, Binder EB (2015) Epigenetics of stress-related psychiatric disorders and gene × environment interactions. Neuron 86:1343–1357. 10.1016/j.neuron.2015.05.036 26087162

[B68] Krugers HJ, Arp JM, Xiong H, Kanatsou S, Lesuis SL, Korosi A, Joels M, Lucassen PJ (2017) Early life adversity: lasting consequences for emotional learning. Neurobiol Stress 6:14–21. 10.1016/j.ynstr.2016.11.005 28229105 PMC5314442

[B69] Kumar G, Jones NC, Morris MJ, Rees S, O'Brien TJ, Salzberg MR (2011) Early life stress enhancement of limbic epileptogenesis in adult rats: mechanistic insights. PLoS One 6:e24033. 10.1371/journal.pone.0024033 21957442 PMC3177819

[B70] Kunugi H, Ida I, Owashi T, Kimura M, Inoue Y, Nakagawa S, Yabana T, Urushibara T, Kanai R, Aihara M, Yuuki N, Otsubo T, Oshima A, Kudo K, Inoue T, Kitaichi Y, Shirakawa O, Isogawa K, Nagayama H, Kamijima K, et al. (2006) Assessment of the dexamethasone/CRH test as a state-dependent marker for hypothalamic- pituitary- adrenal (HPA) axis abnormalities in major depressive episode: a multicenter study. Neuropsychopharmacology 31:212–220. 10.1038/sj.npp.1300868 16123748

[B71] Künzel HE, Binder EB, Nickel T, Ising M, Fuchs B, Majer M, Pfennig A, Ernst G, Kern N, Schmid DA, Uhr M, Holsboer F, Modell S (2003) Pharmacological and non pharmacological factors influencing hypothalamic- pituitary- adrenocortical axis reactivity in acutely depressed psychiatric in patients, measured by the Dex-CRH test. Neuropsychopharmacology 28:2169–2178. 10.1038/sj.npp.1300280 12931142

[B72] Ladd CO, Huot RL, Thrivikraman KV, Nemeroff CB, Meaney MJ, Plotsky PM (1999) Long-term behavioral and neuroendocrine adaptations to adverse early experience Prog Brain Res 122:81–103. 10.1016/s0079-6123(08)62132-9 10737052

[B73] Lembke A, Gomez R, Tenakoon L, Keller J, Cohen G, Williams GH, Kraemer FB, Schatzberg AF (2013) The mineralocorticoid receptor agonist, fludrocortisone, differentially inhibits pituitary-adrenal activity in humans with psychotic major depression. Psychoneuroendocrinology 38:115–121. 10.1016/j.psyneuen.2012.05.006 22727477 PMC3633490

[B74] Lesse A, Rether K, Gröger N, Braun K, Bock J (2017) Chronic postnatal stress induces depressive-like behavior in male mice and programs second-hit stress-induced gene expression patterns of OxtR and AvpR1a in adulthood. Mol Neurobiol 54:4813–4819. 10.1007/s12035-016-0043-8 27525673

[B75] Levitan RD, Vaccarino FJ, Brown GM, Kennedy SH (2002) Low-dose dexamethasone challenge in women in women with atypical major depression. J Psychiatry Neurosci 27:47–51. 11836976 PMC149795

[B76] Loi M, Koricka S, Lucassen P, Joëls M (2014) Age-and sex-dependent effects of early life stress on hippocampal neurogenesis. Front Endocrinol (Lausanne) 5:13. 10.3389/fendo.2014.00013 24600436 PMC3929839

[B77] Lupien SJ, McEwen BS, Gunnar MR, Heim C (2009) Effects of stress throughout the lifespan on the brain, behaviour and cognition. Nat Rev Neurosci 10:434–445. 10.1038/nrn2639 19401723

[B78] Lyons DM, Parker KJ, Schatzberg AF (2010) Animal models of early life stress: implications for understanding resilience. Dev Psychobiol 52:616–624. 10.1002/dev.20500 20957724 PMC6716163

[B79] Macedo BBD, Baes CVW, Menezes IC, Juruena MF (2019) Child abuse and neglect as risk factors for comorbidity between depression and chronic pain in adulthood. J Nerv Ment Dis 207:538–545. 10.1097/NMD.0000000000001031 31192794

[B80] Manly JT, Kim JE, Rogosch FA, Cicchetti D (2001) Dimensions of child maltreatment and children's adjustment: contributions of developmental timing and subtype. Dev Psychopathol 13:759–782. 10.1017/S0954579401004023 11771907

[B81] Martins-Monteverde CMS, Baes CVW, Reisdorfer E, Padovan T, De Carvalho Tofoli SM, Juruena MF (2019) Relationship between depression and subtypes of early life stress in adult psychiatric patients. Front Psychiatry 10:19–18. 10.3389/fpsyt.2019.00019 30804815 PMC6370718

[B82] Mazer AK, Cleare AJ, Young AH, Juruena MF (2019) Bipolar affective disorder and borderline personality disorder: differentiation based on the history of early life stress and psychoneuroendocrine measures. Behav Brain Res 357-358:48–56. 10.1016/j.bbr.2018.04.015 29702176

[B83] Mello AF, Juruena MF, Pariante CM, Tyrka AR, Price LH, Carpenter LL, Del Porto JA (2007) Depressão e estresse: existe um endofenótipo? Rev Bras Psiquiatr 29:s13–s18. 10.1590/S1516-44462007000500004 17546342 PMC4467732

[B84] Mello MF, Faria AA, Mello AF, Carpenter LL, Tyrka AR, Price LH (2009) Childhood maltreatment and adult psychopathology: pathways to hypothalamic-pituitary-adrenal axis dysfunction. Rev Bras Psiquiatr 31. 10.1590/s1516-44462009000600002 PMC4476494 19967199

[B85] Micromedex (2020) IBM Watson health. Greenwood Village, Colorado, USA. Accessed August 27,2020. Available at https://www.micromedexsolutions.com/.

[B86] Mineur YS, Belzung C, Crusio WE (2006) Effects of unpredictable chronic mild stress on anxiety and depression-like behavior in mice. Behav Brain Res 175:43–50. 10.1016/j.bbr.2006.07.029 17023061

[B87] Miragaia AS, de Oliveira Wertheimer GS, Consoli AC, Cabbia R, Longo BM, Girardi CE, Suchecki D (2018) Maternal deprivation increases anxiety-and depressive-like behaviors in an age-dependent fashion and reduces neuropeptide Y expression in the amygdala and hippocampus of male and female young adult rats. Front Behav Neurosci 12:15. 30131681 10.3389/fnbeh.2018.00159 PMC6090069

[B88] Molendijk ML, de Kloet ER (2015) Immobility in the forced swim test is adaptive and does not reflect depression. Psychoneuroendocrinology 62:389–391. 10.1016/j.psyneuen.2015.08.028 26386543

[B89] Molendijk ML, de Kloet ER (2019) Coping with the forced swim stressor: current state-of-the-art. Behav Brain Res 364:1–10. 10.1016/j.bbr.2019.02.005 30738104

[B90] Molet J, Maras PM, Avishai‐Eliner S, Baram TZ (2014) Naturalistic rodent models of chronic early‐life stress. Dev Psychobiol 56:1675–1688. 10.1002/dev.21230 24910169 PMC4777289

[B91] Monroe SM, Harkness KL (2005) Life stress, the “kindling” hypothesis, and the recurrence of depression: considerations from a life stress perspective. Psychol Rev 112:417–445. 10.1037/0033-295X.112.2.417 15783292

[B92] Murmu MS, Salomon S, Biala Y, Weinstock M, Braun K, Bock J (2006) Changes of spine density and dendritic complexity in the prefrontal cortex in offspring of mothers exposed to stress during pregnancy. Eur J Neurosci 24:1477–1487. 10.1111/j.1460-9568.2006.05024.x 16965544

[B93] Naninck EFG, Hoeijmakers L, Kakava-Georgiadou N, Meesters A, Lazic SE, Lucassen PJ, Korosi A (2015) Chronic early life stress alters developmental and adult neurogenesis and impairs cognitive function in mice. Hippocampus 25:309–328. 10.1002/hipo.22374 25269685

[B94] Newport DJ, Heim C, Bonsall R, Miller AH, Nemeroff CB (2004) Pituitary-adrenal responses to standard and low-dose dexamethasone suppression tests in adult survivors of child abuse. Biol Psychiatry 55:10–20. 10.1016/s0006-3223(03)00692-9 14706420

[B95] Norman RE, Byambaa M, De R, Butchart A, Scott J, Vos T (2012) The long-term health consequences of child physical abuse, emotional abuse, and neglect: a systematic review and meta-analysis. PLoS Med 9:e1001349. 10.1371/journal.pmed.1001349 23209385 PMC3507962

[B96] Novais A, Monteiro S, Roque S, Correia-Neves M, Sousa N (2017) How age, sex and genotype shape the stress response. Neurobiol Stress 6:44–56. 10.1016/j.ynstr.2016.11.004 28229108 PMC5314441

[B97] Otte C, Hinkelmann K, Moritz S, Yassouridis A, Jahn H, Wiedemann K, Kellner M (2010) Modulation of the mineralocorticoid receptor as add-on treatment in depression: a randomized, double-blind, placebo-controlled proof-of-concept study. J Psychiatr Res 44:339–346. 10.1016/j.jpsychires.2009.10.006 19909979

[B98] Papp M, Willner P, Muscat R, Street OC (1991) By chronic unpredictable mild stress. Psychopharmacology (Berl) 104:255–259. 10.1007/BF02244188 1876670

[B99] Pariante CM, Papadopoulos AS, Poon L, Checkley SA, English J, Kerwin RW, Lightman S (2002) A novel prednisolone suppression test for the hypothalamic-pituitary-adrenal axis. Biol Psychiatry 51:922–930. 10.1016/s0006-3223(01)01314-2 12022966

[B100] Paslakis G, Krumm B, Gilles M, Schweiger U, Heuser I, Richter I, Deuschle M (2011) Discrimination between patients with melancholic depression and healthy controls: comparison between 24-h cortisol profiles, the DST and the Dex/CRH test. Psychoneuroendocrinology 36:691–698. 10.1016/j.psyneuen.2010.10.002 21035272

[B101] Pintor L, Torres X, Navarro V, Martinez de Osaba MJ, Matrai S, Gastó C (2007) Corticotropin-releasing factor test in melancholic patients in depressed state versus recovery: a comparative study. Prog Neuropsychopharmacol Biol Psychiatry 31:1027–1033. 10.1016/j.pnpbp.2007.03.002 17433515

[B102] Pompili M, Iliceto P, Innamorati M, Rihmer Z, Lester D, Akiskal HS, Girardi P, Ferracuti S, Tatarelli R (2009) Suicide risk and personality traits in physically and/or sexually abused acute psychiatric inpatients: a preliminary study. Psychol Rep 105:554–568. 10.2466/PR0.105.2.554-568 19928616

[B103] Porsolt RD, Le P, Jalfre ML (1977) Depression: a new animal model sensitive to antidepressant treatments. Nature 266:730–732. 10.1038/266730a0 559941

[B104] Post RM (2002) Do the epilepsies, pain syndromes, and affective disorders share common kindling-like mechanisms? Epilepsy Res 50:203–219. 10.1016/S0920-1211(02)00081-5 12151130

[B105] Reul JM, Bilang-Bleuel A, Droste S, Linthorst ACE, Holsboer F, Gesing A (2000) New mode of hypothalamic-pituitary-adrenocortical axis regulation: significance for stress-related disorders. Z Rheumatol 59:II/22–5. 10.1007/s003930070013 11155799

[B106] Rice CJ, Sandman CA, Lenjavi MR, Baram TZ (2008) A novel mouse model for acute and long-lasting consequences of early life stress. Endocrinology 149:4892–4900. 10.1210/en.2008-0633 18566122 PMC2582918

[B107] Rogerson FM, Yao YZ, Smith BJ, Dimopoulos N, Fuller PJ (2003) Determinants of spironolactone binding specificity in the mineralocorticoid receptor. J Mol Endocrinol 31:573–582. 10.1677/jme.0.0310573 14664717

[B108] Rush AJ, Trivedi MH, Wisniewski SR, Nierenberg AA, Stewart JW, Warden D, Niederehe G, Thase ME, Lavori PW, Lebowitz BD, McGrath PJ, Rosenbaum JF, Sackeim HA, Kupfer DJ, Luther J, Fava M (2006) Acute and longer-term outcomes in depressed outpatients requiring one or several treatment steps: a STAR*D report. Am J Psychiatry 163:1905–1917. 10.1176/ajp.2006.163.11.1905 17074942

[B109] Samtani MN, Jusko WJ (2005) Comparison of dexamethasone pharmacokinetics in female rats after intravenous and intramuscular administration. Biopharm Drug Dispos 26:85–91. 10.1002/bdd.435 15654687 PMC4178533

[B110] Samtani MN, Jusko WJ (2007) Quantification of dexamethasone and corticosterone in rat biofluids and fetal tissue using highly sensitive analytical methods: assay validation and application to pharmacokinetic study. Biomed Chromatogr 21:585–597. 10.1002/bmc.788 17385808 PMC4183228

[B111] Schane HP, Potts GO (1978) Oral progestational activity of spironolactone. J Clin Endocrinol Metab 47:691–694. 10.1210/jcem-47-3-691 95623

[B112] Schwartz AC, Bradley RL, Sexton M, Sherry A, Ressler KJ (2005) Posttraumatic stress disorder among African Americans in an inner city mental health clinic. Psychiatr Serv 56:212–215. 10.1176/appi.ps.56.2.212 15703352

[B113] Sfoggia A, Pacheco MA, Grassi-Oliveira R (2008) History of childhood abuse and neglect and suicidal behavior at hospital admission. Crisis 29:154–158. 10.1027/0227-5910.29.3.154 18714912

[B114] Shapero BG, Black SK, Liu RT, Klugman J, Bender RE, Abramson LY, Alloy LB (2014) Stressful life events and depression symptoms: the effect of childhood emotional abuse on stress reactivity. J Clin Psychol 70:209–223. 10.1002/jclp.22011 23800893 PMC3884028

[B115] Sheehan DV, Lecrubier Y, Sheehan KH, Amorim P, Janavs J, Weiller E, Hergueta T, Baker R, Dunbar GC (1998) The Mini-International Neuropsychiatric Interview (M.I.N.I.): the development and validation of a structured diagnostic psychiatric interview for DSM-IV and ICD-10. J Clin Psychiatry 59:22–33. 9881538

[B116] Slattery DA, Cryan JF (2012) Using the rat forced swim test to assess antidepressant-like activity in rodents. Nat Protoc 7:1009–1014. 10.1038/nprot.2012.044 22555240

[B117] Stetler C, Miller GE (2011) Depression and hypothalamic-pituitary-adrenal activation: a quantitative summary of four decades of research. Psychosom Med 73:114–126. 10.1097/PSY.0b013e31820ad12b 21257974

[B118] Spitzer C, Otte C, Kuehl LK, May A, Schultebraucks K, Hellmann-Regen J, Wingenfeld K (2018) The dexamethasone corticotropin releasing hormone test in healthy and depressed women with and without childhood adversity. Psychoneuroendocrinology 87:147–151. 10.1016/j.psyneuen.2017.10.016 29080551

[B119] Straus MA, Hamby SL, Finkelhor D, Moore DW, Runyan D (1998) Identification of child maltreatment with the parent-child conflict tactics scales: development and psychometric data for a national sample of American parents. Child Abus Negl 22:249–270. 10.1016/S0145-2134(97)00174-9 9589178

[B120] Tunnard C, Rane LJ, Wooderson SC, Markopoulou K, Poon L, Fekadu A, Juruena M, Cleare AJ (2014) The impact of childhood adversity on suicidality and clinical course in treatment-resistant depression. J Affect Disord 152–154:122–130. 10.1016/j.jad.2013.06.037 23880448

[B121] Tyrka AR, Wier L, Price LH, Ross N, Anderson GM, Wilkinson CW, Carpenter LL (2008) Childhood parental loss and adult hypothalamic-pituitary-adrenal function. Biol Psychiatry 63:1147–1154. 10.1016/j.biopsych.2008.01.011 18339361 PMC2650434

[B122] Umeoka EHL, Garcia SB, Antunes-Rodrigues J, Elias LLK, Garcia-Cairasco N (2011) Functional characterization of the hypothalamic-pituitary-adrenal axis of the Wistar audiogenic rat (WAR) strain. Brain Res 1381:141–147. 10.1016/j.brainres.2011.01.042 21256829

[B123] Walker CD, Bath KG, Joels M, Korosi A, Larauche M, Lucassen PJ, Morris MJ, Raineki C, Roth TL, Sullivan RM, Taché Y, Taché Y (2017) Chronic early life stress induced by limited bedding and nesting (LBN) material in rodents: critical considerations of methodology, outcomes and translational potential. Stress 20:421–448. 10.1080/10253890.2017.1343296 28617197 PMC5705407

[B124] Weaver IC, Cervoni N, Champagne FA, D'Alessio AC, Sharma S, Seckl JR, Dymov S, Szyf M, Meaney MJ (2004) Epigenetic programming by maternal behavior. Nat neurosci 7:847–854. 10.1038/nn1276 15220929

[B125] Williams LM, Debattista C, Duchemin AM, Schatzberg AF, Nemeroff CB (2016) Childhood trauma predicts antidepressant response in adults with major depression: data from the randomized international study to predict optimized treatment for depression. Transl Psychiatry 6:e799–e799. 10.1038/tp.2016.61 27138798 PMC5070060

[B126] Wright KD, Asmundson GJG, McCreary DR, Scher C, Hami S, Stein MB (2001) Factorial validity of the childhood trauma questionnaire in men and women. Depress Anxiety 13:179–183. 11413564

[B127] Zhang J, Abdallah CG, Chen Y, Huang T, Huang Q, Xu C, Xiao Y, Liu Y, Ding Y, Wu R (2013) Behavioral deficits, abnormal corticosterone, and reduced prefrontal metabolites of adolescent rats subject to early life stress. Neurosci Lett 545:132–137. 10.1016/j.neulet.2013.04.035 23643993 PMC3699722

